# In Silico and In Vitro Evaluation of the Antimicrobial Potential of *Bacillus cereus* Isolated from *Apis dorsata* Gut against *Neisseria gonorrhoeae*

**DOI:** 10.3390/antibiotics10111401

**Published:** 2021-11-15

**Authors:** Nurdjannah Jane Niode, Aryani Adji, Jimmy Rimbing, Max Tulung, Mohammed Alorabi, Ahmed M. El-Shehawi, Rinaldi Idroes, Ismail Celik, Fatimawali, Ahmad Akroman Adam, Kuldeep Dhama, Gomaa Mostafa-Hedeab, Amany Abdel-Rahman Mohamed, Trina Ekawati Tallei, Talha Bin Emran

**Affiliations:** 1 Entomology Study Program, Graduate School, University of Sam Ratulangi. Jl. Kampus Unsrat, Manado 95115, North Sulawesi, Indonesia; niodejane@unsrat.ac.id (N.J.N.); aryaniadji@gmail.com (A.A.); jimmyrimbing@unsrat.ac.id (J.R.); tulungmax@unsrat.ac.id (M.T.); 2 Department of Dermatology and Venereology, Faculty of Medicine, University of Sam Ratulangi, RD Kandou Hospital, Jl. Raya Tanawangko No. 56, Manado 95163, North Sulawesi, Indonesia; 3 Department of Biotechnology, College of Science, Taif University, P.O. Box 11099, Taif 21944, Saudi Arabia; maorabi@tu.edu.sa (M.A.); elshehawi@hotmail.com (A.M.E.-S.); 4 Department of Pharmacy, Faculty of Mathematics and Natural Sciences, University of Syiah Kuala, Kopelma Darussalam, Banda Aceh 23111, Aceh, Indonesia; rinaldi.idroes@unsyiah.ac.id; 5 Department of Chemistry, Faculty of Mathematics and Natural Sciences, Universitas Syiah Kuala, Banda Aceh 23111, Aceh, Indonesia; 6 Department of Pharmaceutical Chemistry, Faculty of Pharmacy, Erciyes University, Kayseri 38039, Turkey; ismailcelik@erciyes.edu.tr; 7 Pharmacy Study Program, Faculty of Mathematics and Natural Sciences, University of Sam Ratulangi, Manado 95115, North Sulawesi, Indonesia; fatimawali@unsrat.ac.id; 8 Dentistry Study Program, Faculty of Medicine, University of Sam Ratulangi, Manado 95115, North Sulawesi, Indonesia; ahmad_adam@ymail.com; 9 Division of Pathology, ICAR-Indian Veterinary Research Institute, Izatnagar, Bareilly 243 122, Uttar Pradesh, India; kdhama@rediffmail.com; 10 Pharmacology Department, Health Sciences Research Unit, Medical College, Jouf University, Skaka 11564, Saudi Arabia; gomaa@ju.edu.sa; 11 Pharmacology Department, Faculty of Medicine, Beni-Suef University, Beni-Suef 62521, Egypt; 12 Department of Forensic Medicine and Toxicology, Zagazig University, Zagazig 4511, Egypt; aabdaziz@zu.edu.eg; 13 Department of Biology, Faculty of Mathematics and Natural Sciences, University of Sam Ratulangi, Manado 95115, North Sulawesi, Indonesia; 14 Department of Pharmacy, BGC Trust University Bangladesh, Chittagong 4381, Bangladesh

**Keywords:** lipopeptide, *Apis dorsata*, *Bacillus cereus*, *Neisseria gonorrhoeae*, insect gut, antimicrobial activity

## Abstract

Antimicrobial resistance is a major public health and development concern on a global scale. The increasing resistance of the pathogenic bacteria *Neisseria gonorrhoeae* to antibiotics necessitates efforts to identify potential alternative antibiotics from nature, including insects, which are already recognized as a source of natural antibiotics by the scientific community. This study aimed to determine the potential of components of gut-associated bacteria isolated from *Apis dorsata*, an Asian giant honeybee, as an antibacterial against *N. gonorrhoeae* by in vitro and in silico methods as an initial process in the stage of new drug discovery. The identified gut-associated bacteria of *A. dorsata* included *Acinetobacter indicus* and *Bacillus cereus* with 100% identity to referenced bacteria from GenBank. Cell-free culture supernatants (CFCS) of *B. cereus* had a very strong antibacterial activity against *N. gonorrhoeae* in an in vitro antibacterial testing. Meanwhile, molecular docking revealed that antimicrobial lipopeptides from *B. cereus* (surfactin, fengycin, and iturin A) had a comparable value of binding-free energy (BFE) with the target protein receptor for *N. gonorrhoeae*, namely penicillin-binding protein (PBP) 1 and PBP2 when compared with the ceftriaxone, cefixime, and doxycycline. The molecular dynamics simulation (MDS) study revealed that the surfactin remains stable at the active site of PBP2 despite the alteration of the H-bond and hydrophobic interactions. According to this finding, surfactin has the greatest antibacterial potential against PBP2 of *N. gonorrhoeae*.

## 1. Introduction

Gonorrhea is a sexually transmitted infection (STI) caused by *Neisseria gonorrhoeae*, a Gram-negative, coffee-bean-shaped facultative intracellular diplococcus bacterium [1,2]. The global prevalence of urogenital gonorrhea in 2016 was estimated at 0.9% in women and 0.7% in men, corresponding to a total of 30.6 million worldwide gonorrhea cases [3]. According to data from 13 teaching hospitals in Indonesia from 2015 to 2017, gonorrhea is the third most common sexually transmitted infection (STI), based on the data released by the Indonesian Sexually Transmitted Infection Study Group in 2018.

Until now, gonorrhea has been a source of concern due to the complications it causes, including infertility in men and women, ectopic pregnancy caused by scar tissue formation in the fallopian tubes, and blindness [4,5,6]. Another significant issue is the discovery of resistant *N. gonorrhoeae* strains in several countries, as well as the failure of therapy [7,8]. The Centers for Disease Control and Prevention (CDC) considers gonorrhea treatment as an ‘urgent threat’ due to the fact that eighteen medicines have acquired resistance in the United States. The World Health Organization (WHO) has designated *N. gonorrhoeae* as a high-priority pathogen for antibiotic research and development [9]. Numerous efforts have been made to address the spread and impact of antimicrobial *N. gonorrhoeae* resistance, including research into alternative therapeutic regimes [10,11,12].

Nowadays, efforts to discover new antibacterial drugs derived from nature are attracting the attention of researchers worldwide, including those studying insects. The honey bee is one of the insects that researchers are beginning to study in their search for new drugs [13,14]. *Apis dorsata* is an Asian giant honeybee species that prefers to live in forests. It is found throughout Indonesia, with the exception of Maluku and Irian Jaya [15]. Of the three subspecies of *A. dorsata*, two of them are found in Indonesia, namely *A. dorsata dorsata* and *A. dorsata binghami*, while the third subspecies, *A. dorsata breviligula*, is found in the Philippines [16].

In general, *Apis* is an insect that is not only beneficial as a pollinator and honey producer but also has the potential to be a source of bioactive compounds in the health sector [17,18]. As a social insect that lives in colonies with a food-sharing system and a close relationship between colony members, honeybees have a unique source of microbes [19].

The Apis gut microbial community is composed of three major phyla: Proteobacteria, Firmicutes, and Actinobacteria [20,21]. Among the bacteria belonging to these phyla, there are several species of lactic acid bacteria (LAB) [22,23,24]. LAB has potential as a probiotic because the bioactive components it produces are synergistic with antimicrobials [25]. Probiotics, according to the Food and Agricultural Organization (FAO) and the World Health Organization (WHO), are live microorganisms that, when administered in sufficient quantities, can provide health benefits to the host [26].

Nowadays, the use of probiotics derived from unusual sources, such as animal digestive tracts, is on the rise [27]. Several LABs isolated from the gut of *Apis*, such as *Lactobacillus*, *Bifidobacterium*, and *Enterococcus*, showed inhibitory effects against pathogenic bacteria [22,24], indicating their potential as probiotics. Antimicrobial peptide, one of the bioactive compounds of LAB from *Apis* gut, is a critical component of bee immune systems and has the potential to be developed as a broad-spectrum antibiotic for treating or preventing bacterial infections [28,29,30].

The genus *Bacillus* is one of the bacteria that can be found in honey [31] and honeybee larval gut [32,33]. The bacterium is capable of producing a large number of antimicrobial peptides [34]. *Bacillus cereus* is one of the *Bacillus* species that has the ability to produce antimicrobial peptides [35,36]. According to Chauhan et al. [37], *B. cereus* TSH77 is capable of producing surfactin and fengycin. The complete genome sequence of *B. cereus* strain ATCC 14579 reveals that this bacterium’s genome contains a chromosomal gene cluster that may code for the biosynthesis of a novel peptide antibiotic [38]. Given the critical nature of developing novel anti-gonococcal regimens, this study was aimed at investigating the antimicrobial potential of gut-associated bacteria from *A. dorsata* as a potential source of new antibiotics against *N. gonorrhoeae*. This study combines in vitro and in silico tests. Adoption of in vitro and in silico alternatives to animal testing in pharmaceutical drug development is opening up new avenues for improving success rates and shortening drug discovery times [39,40]. The use of in silico approaches in regulatory decision-making will increase as public confidence in their applicability and reliability grows [41].

## 2. Results

### 2.1. In Vitro Antibacterial Assay

In this study, the antibacterial activity of bacteria isolated from *A. dorsata* gut was tested against the pathogenic bacterium *N. gonorrhoeae*. The antibiotic doxycycline served as a positive control, while sterile ddH_2_0 served as a negative control. Table 1 shows the antibacterial test results of each isolate’s supernatant against *N. gonorrhoeae*. According to the findings, four isolates (LJ2, LJ4, LJ5, and LJ7) had strong antibacterial activity, and one isolate (LJ6) had very strong antibacterial activity in treatment 1 (heat-killed at 80 °C). According to the antibacterial test results of the supernatant of each isolate neutralized with NaOH (treatment 2), three isolates (LJ2, LJ5, and LJ7) had a strong antibacterial activity, while two isolates (LJ4 and LJ6) had very strong antibacterial activity,. In treatment 2, four isolates (LJ2, LJ4, LJ6, and LJ7) demonstrated greater activity than the isolates in treatment 1. Isolate LJ6 produced the highest antibacterial activity in treatment 1, while isolate LJ4 produced the highest antibacterial activity in treatment 2.

### 2.2. Molecular Identification of the Isolates

All bacterial isolates were identified using the 16S rRNA gene as a molecular marker (Table 2). The isolate LJ1 shared 99.92% identity with *B. anthracis*, *B. thuringiensis*, *B. cereus*, *B. tropicus*, and *B. paramycoides*. Isolate LJ2 was identified as *Acinetobacter indicus*. Isolates LJ3, LJ6, and LJ7 were identified as *B. cereus*. Isolates LJ4 and LJ5 could not be identified.

### 2.3. Molecular Docking Study of the Lipopeptide of Bacillus

**Figure 1:**
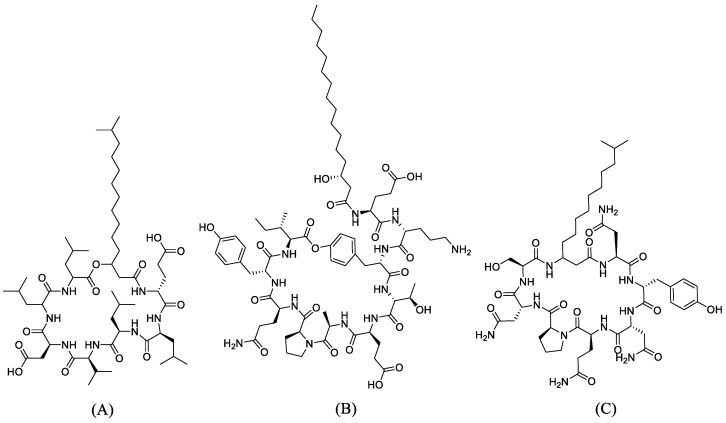
The 2D structures of the ligands: (**A**) Surfactin, (**B**) Fengycin, and (**C**) Iturin A.

Surfactin, fengycin, and iturin A were chosen as ligands for molecular docking in this present study. The 2D structures of surfactin, fengycin, and iturin A (Figure 1A–C) were downloaded from the PubChem database page to be used as ligands in the molecular docking process. As a positive control for the ligand, cefixime and ceftriaxone were used as the treatments of choice for gonorrhea, as well as doxycycline, which is usually given together to treat multiple infectious urethritis/cervicitis. *N. gonorrhoeae* has four penicillin-binding proteins (PBPs). PBP1 and 2 were used as receptors, both of which were downloaded from the PDB website.

The results of the molecular docking analysis indicated that each ligand, particularly fengycin and surfactin, had a strong interaction with the receptor (Table 3). The binding free energy of the ligands to the PBP1 receptor varied between −103.21 and −124.42 kcal/mol. Iturin A had the lowest value (−124.42 kcal/mol), followed by surfactin (−118.37), then fengycin (−103.21 kcal/mol), in comparison to ceftriaxone (−117.49 kcal/mol), cefixime (−105.15 kcal/mol), and doxycycline (−104.23 kcal/mol). While the binding free energy of the ligands to the PBP2 receptor varied between −103.74 and 137.87 kcal/mol. Iturin A produced a value of −127.67 kcal/mol, followed by fengycin (−114.55 kcal/mol) and surfactin (−103.74), compared with ceftriaxone (−137.87 kcal/mol), cefixime (−113.39 kcal/mol), and doxycycline (−113.13 kcal/mol). 

**Figure 2:**
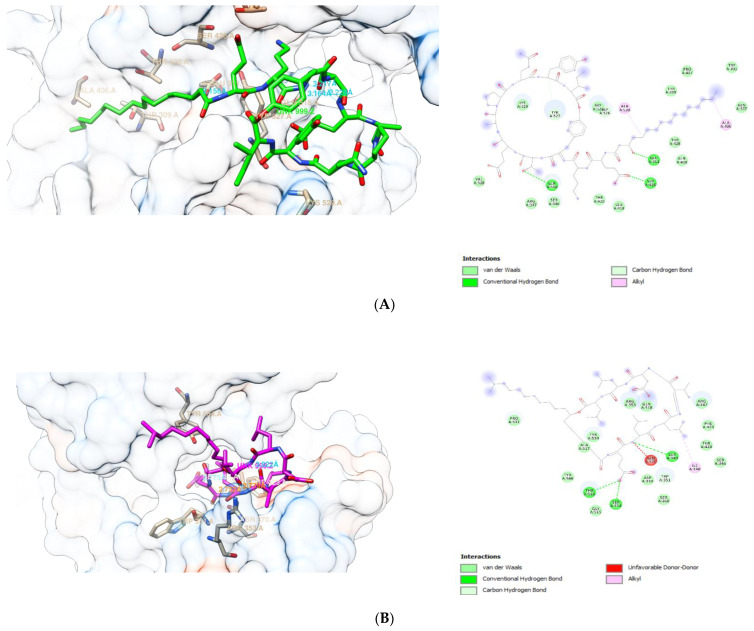

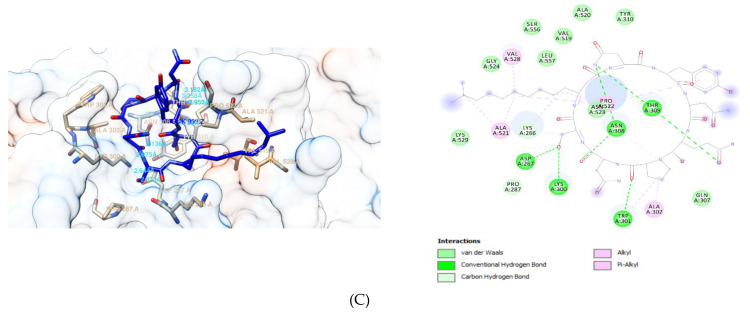
Molecular interaction between PBP1 with (**A**) fengycin, (**B**) surfactin, and (**C**) iturin A.

**Figure 3:**
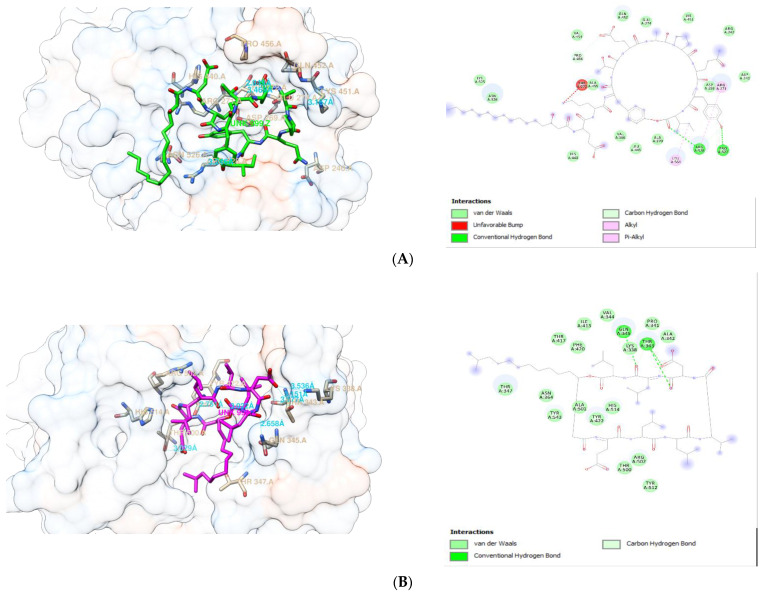

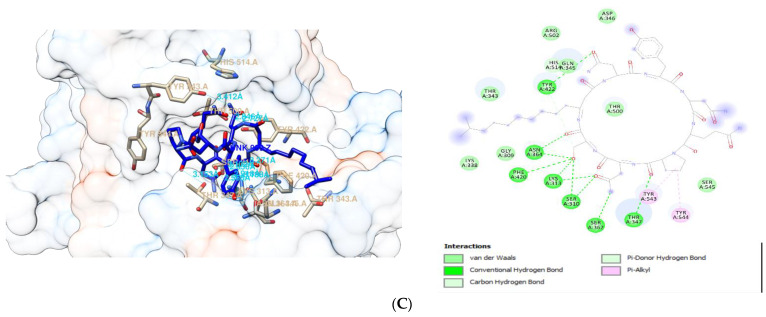
Molecular interaction between PBP2 with (**A**) fengycin, (**B**) surfactin, and (**C**) iturin A.

The visualization of the interaction between fengycin, surfactin, and iturin A with PBP 1 and 2, respectively, is shown in Figure 2 and Figure 3. These compounds’ interactions with the active sites of the receptors are stabilized by H-bonds and non-bonded interactions (Table 4 and Table 5). Each ligand exhibited multiple interactions with its receptor, while also forming hydrogen bonds with the receptor. At PBP1, fengycin forms three conventional H-bonds with Gln:A518, Glu:A311, and Ser:A426, one carbon H-bonds with Tyr:A527, and two alkyl bonds with Ala:A520,Ala:A406. Surfactin forms three H-bonds at PBP1 with Ser:A349, Thr:A516, and Ser:A314, one carbon H-bond with Trp:A351, and one alkyl bond with Ile:A348. Iturin A forms seven conventional H-bonds at PBP1 with two Thr:A309, two Asn:A308, Trp:A301,Asp:A267, Lys:A300, two carbon H-bonds with Asn:A308 and Lys:A266, and alkyl/pi-Alkyl interactions with two Ala:A521, Lys:A266, Val:A528, three Pro:A522, Trp:A301, and Ala:A302. Apparently, the binding sites of fengycin, surfactin, and iturin A at PBP1 are not the same as those of ceftriaxone, cefixime, and doxycycline.

At PBP2, fengycin forms conventional H-bonds with Arg:A528 and Pro:A522, carbon H-bonds with Arg:A528 and Pro:A456, alkyl/pi-Alkyl bonds with Arg:A271, Arg:A528, and Leu:A564, as well as an unfavorable bump with Thr:A272. Surfactin forms conventional H-bond with two Thr:A343 and Gln:A345, as well as carbon H-bond with Thr:A343 at PBP2. Meanwhile, at PBP2, iturin A forms conventional H-bonds with two Asn:A364, Phe:A420, two LysA313, two Ser:A310, Ser:A362, Thr:A347, and Tyr:A422, carbon H-bond with Tyr:422;, and Pi-Alkyl with Tyr:A543 and Tyr:A544. Another point to mention is that the binding sites for fengycin, surfactin, and iturin A at PBP2 are distinct from the binding sites for ceftriaxone, cefixime, and doxycycline.

### 2.4. Molecular Dynamics Simulation Study

**Figure 4:**
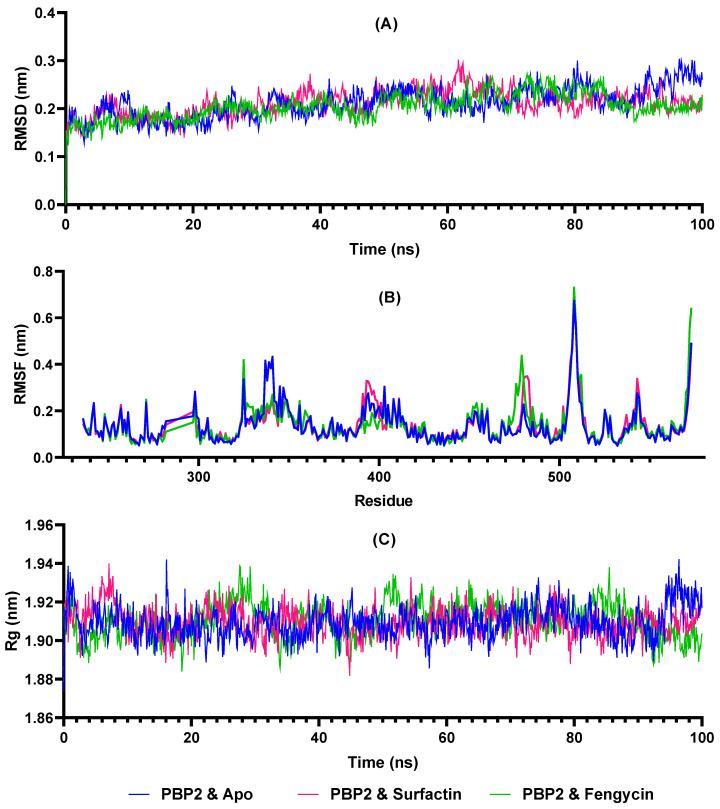
Molecular dynamics simulations of apoprotein (PBP2 and Apo), Surfactin (PBP2 and Surfactin) and Fengycin (PBP2 and Fengycin) complexes with penicillin-binding protein 2 (PBP2) (**A**) RMSD of apoprotein, Surfactin and Fengycin bound PBP2 complexes, (**B**) RMS fluctuation, and (**C**) Rg plots during the period of 100 ns simulation.

Molecular dynamics simulations are required to ascertain the stability of the interaction between the two compounds (fengycin and surfactin) and the receptors PBP 1 and 2. As a result, MD simulations were run for 100 ns in this present study of PBP2–fengycin and PBP2–surfactin complexes. Figure 4 demonstrates that the PBP2–surfactin and PBP2–fengycin complexes remained stable throughout time. The RMSD value of the complex was approximately below 0.3 nm, and average RMSD values of 0.211 nm for the apoprotein, 2.210 nm for the PBP2–surfactin complex, and 0.204 nm for the PBP2–fengycin complex were measured. As given in the RMSF plot in Figure 4, because surfactin and fengycin form H-bonds with Pro341, Thr343, and Gln345 at the PBP2 binding site, these amino acids fluctuate less around these amino acids than apoprotein. At other residues, the apoprotein and holoproteins exhibited similar RMSF profiles. Rg values remained constant, fluctuating between 1.88 and 1.95 nm. The PBP2 apoprotein gave average Rg values of 1.909 nm, the PBP2–surfactin complex 1.909 nm, and the PBP2–fengycin complex 1.910 nm.

### 2.5. MM-PBSA Calculations

**Figure 5:**
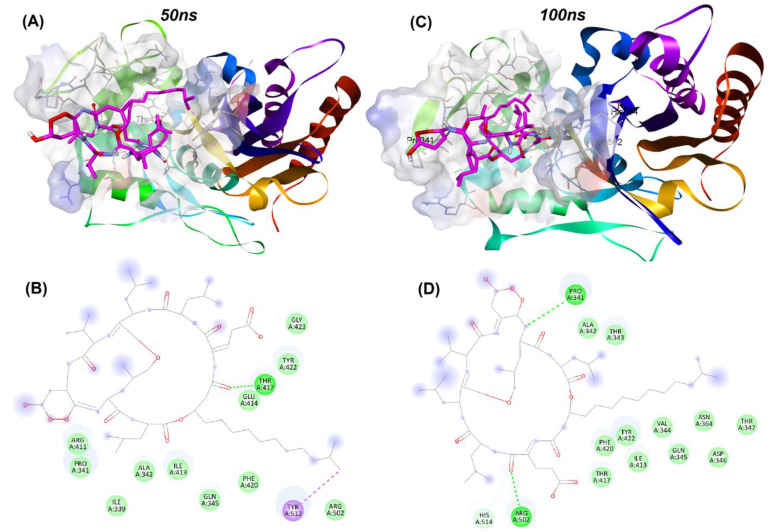
Molecular interactions of surfactin at 50 ns and 100 ns at PBP2 active site (**A**) binding pose of surfactin at 50 ns and (**B**) schematic protein–ligand interaction diagram of the binding of surfactin and PBP2 active site at 50 ns simulation, (**C**) binding pose of surfactin at 100 ns and (**D**) schematic protein–ligand interaction diagram of the binding of surfactin and PBP2 active site at 100 ns simulation.

Table 6 showed the BFE value based on MM-PBSA calculation of PBP2 with surfactin and fengycin between 80 ns and 100 ns. The calculated interactions between PBP2 and surfactin were slightly stronger (124.564 kJ/mol) than those between PBP2 and fengycin (−115.557 kJ/mol). According to the MM-PBSA calculation, the protein–ligand interactions and binding pose of the surfactin compound, which has a higher interaction with PBP2, were analyzed at 50 ns and 100 ns. As shown in Figure 5, the surfactin compound remains stable at the active site for up to 100 ns despite the alteration of the H-bond and hydrophobic interactions.

### 2.6. Lipinski’s Rule of Five Analysis

When evaluating a drug candidate, Lipinski’s rule of five (Ro5) should be considered, which includes the following: (1) fewer than five hydrogen bond donors, (2) fewer than ten hydrogen bond acceptors, (3) molecular mass less than 500 Daltons, and (4) log P not greater than 5 [42,43]. The Ro5 analysis of fengycin, surfactin, and iturin A is summarized in Table 7. Fengycin, surfactin, and iturin A appear to violate Ro5.

### 2.7. ADMET Analysis

Adsorption, distribution, metabolism, excretion, and toxicity (ADMET) properties of a compound play critical roles in drug discovery and development. Table 8 shows the results of the analysis. The molecular weights of the compounds under investigation are mostly greater than 500 g/mol, which is in violation of Lipinski’s rule of five (Ro5) [42], except for cefixime and doxycycline. In addition, the H-bond acceptor should not exceed ten. The only compound that qualifies is doxycycline. Moreover, the donor’s H-bond should not exceed five, of which only ceftriaxone and cefixime are eligible.

In addition, a number of other parameters were investigated, including carcinogenicity, hepatotoxicity, central nervous system (CNS) permeability, cytochrome P450 (CYP) inhibition, and acute oral toxicity, among other things. The following is a description of the level of toxicity: Class I (fatal if swallowed, LD50 ≤ 5 mg/kg), Class II (fatal if swallowed, LD50 5 < LD50 ≤ 50 mg/kg), Class III (toxic if swallowed, LD50 50 < LD50 ≤ 300 mg/kg), Class IV (harmful if swallowed, LD50 300 < LD50 ≤ 2000 mg/kg), Class V (maybe harmful if swallowed, LD50 2000 < LD50 ≤ 5000 mg/kg), and Class VI (non-toxic, LD50 > 5000 mg/kg). Almost all of them (with the exception of fengycin, which is in category V) appear to be in category IV, which is harmful if swallowed.

## 3. Discussion

Microbial resistance to antibiotics continues to be a problem in the medical world, indicating the critical need for alternative regimes, particularly those derived from nature. This present study focused on identifying antibacterial agents against *N. gonorrhoeae*. These antibacterial agents are derived from bacteria isolated from the gut of *A. dorsata*. Honey bees have become a concern in the medical world because they are a potential source of antimicrobials. Secondary metabolites produced by bacteria found in honey bees are a source of natural compounds [44].

Doxycycline is a second-generation tetracycline and a low-cost, broad-spectrum antimicrobial agent that is primarily used to treat a variety of bacterial infections, most notably those caused by intracellular pathogens, as well as the bacteria that cause STIs, including *N. gonorrhoeae* [45,46]. The use of doxycycline in this study is consistent with WHO and Indonesian Ministry of Health guidelines, which state that the treatment regimen for gonococcal urethritis and cervicitis should consist of either cefixime 400 mg orally in a single dose, or ceftriaxone 500 mg intramuscular injection given in combination with doxycycline or azithromycin to treat nongonococcal infections that frequently co-occur. This is also because the sensitivity test results revealed that *N. gonorrhoeae* is sensitive to doxycycline. Meanwhile, this bacterium is ceftriaxone and cefixime resistant.

A study has shown that vaginal *Lactobacilli* were able to inhibit the growth of *N. gonorrhoeae* through in vitro studies [47]. According to our finding, gut-associated bacteria isolated from *A. dorsata* exhibited promising antibacterial activity against *N. gonorrhoeae*. This suggests that these bacteria produce substances that can prevent *N. gonorrhoeae* from growing. This is also supported by the research of Ruiz et al. [48], who demonstrated that bacteriocins and other bioactive substances from Lactobacilli exhibited significant inhibitory activity against all gonococci. This means that it is possible to develop bacterial metabolites as candidates for active compounds that inhibit *N. gonorrhoeae*.

Over the last few decades, bacterial identification based on ribosomal RNA genes has long been considered the gold standard for molecular taxonomic study [49,50]. Three isolates showing strong and very strong activities against *N. gonorrhoeae* were identified molecularly as *B. cereus*, while one isolate was identified as *A. indicus*, which shared 100% identity with the reference bacteria in GenBank. Similar results for the activity of *B. cereus* were reported by Lombogia et al. [51], who found that *B. cereus* from the gut of *A. nigrocincta* had antibacterial effects against *S. aureus* and *Escherichia coli*. In their heat-inactivated form, various strains of bacteria, such as lactic acid bacteria and bifidobacteria, can produce beneficial effects [52].

Because the supernatant in treatment 1 was heated, it was assumed that any antimicrobial peptides present would become inactive [53], leaving organic acids, hydrogen peroxide (H_2_O_2_), and alcohol as the bioactive components that inhibited *N. gonorrhoeae*. A recent study demonstrated that probiotic *Lactiplantibacillus plantarum* strains isolated from spontaneously fermented cocoa may possess antimicrobial activity against *N. gonorrhoeae* [54]. Additionally, this probiotic was discovered to produce H_2_O_2_. Another study discovered that H_2_O_2_ produced during the metabolic process has the ability to inhibit bacteria [55]. The findings of Shokryazdan et al. [56] are significant because they show that the antimicrobial activity of CFCS from *Lactobacillus* strains is caused by organic acids. These substances have an antimicrobial mechanism that involves lowering the pH [57].

On the other hand, in treatment 2 supernatant, if there is organic acid present, it will be neutralized. As a result, the bioactive components of antimicrobial peptides and fatty acids contribute to antibacterial activity. Georgieva et al. [58] reported that after pH neutralization, some probiotic strains retained activity, indicating the presence of the active substance. The fact that *B. cereus* LJ6 demonstrated significant antibacterial activity in treatments 1 and 2 indicates that organic acids, H_2_O_2_, and antimicrobial peptides contribute to this activity, making it a candidate for development as a next-generation anti-gonococcal.

Antimicrobial peptides may be active against a broad range of bacteria in generally non-toxic amounts to mammalian cells [59]. Antimicrobial peptides have been shown to kill target cells by interacting with and destabilizing the membrane, leading to depolarization and cell death [60]. These findings imply that peptide-based antimicrobials may evade multiple drug resistance mechanisms [61]. As a result, they may be a more advantageous alternative to conventional antibiotics [62].

*Bacillus* polypeptides with antibacterial properties provide important research results. *B. cereus* TSH77, which can produce surfactin and fengycin, is one of the *Bacillus* species that can produce antibiotics [37]. Furthermore, antibacterial polypeptides produced by *Bacillus* used in medicine include bacitracin, gramicidin S, polymyxin, and tyrothricin [63].

*Bacillus* spp. have been evaluated in vitro and in vivo for their probiotic potential. Several of them exhibit increased acid tolerance and are more resistant to heating and freezing [64], possess immunomodulatory properties [65], antimicrobial [66], and can be used in the fermentation of food [67]. The genus *Acinetobacter* belongs to the family Moraxellaceae, with 61 species that have been published, including *A. indicus*. According to a report, a new strain of *Acinetobacter* KUO 11TH may have the potential to increase resistance to diseases critical to the sustainability of catfish culture [68]. Other studies related to the antibacterial effect of Acinetobacter have not been found.

The antibacterial activity of CFCS from *A. dorsata* gut-associated bacteria was also evaluated in silico using the molecular docking method. A literature review determined that *Bacillus* can produce secondary metabolites with a broad spectrum of antibiotic activity. Surfactin and fengycin are produced by *B. cereus* TSH77, whereas *B. endophyticus* produces surfactin, fengycin, and iturin [37]. These three substances are known as antimicrobial lipopeptides.

Antibacterial lipopeptides, in general, work by damaging the bacterial cell wall [34] and inhibiting the growth of bacterial resistance mechanisms [62]. Surfactin impairs the permeability of the cell membrane [69]. Fengycin has little influence on bulk bilayer order. However, it has a local disrupting effect [70]. Iturin A, on the other hand, has a cytotoxic effect on bacterial plasma membranes [71]. Fengycin and iturin both cause pores in the plasma membrane [72].

The primary molecular target for β-lactam antibiotics used to treat gonococcal infections is PBP2 from *N. gonorrhoeae* [73]. Antibiotics used to treat *N. gonorrhoeae* target peptidoglycan by inhibiting the activity of the essential biosynthetic enzymes PBP1 and PBP2 [74], because PBPs are enzymes that catalyze the final steps of peptidoglycan biosynthesis.

The analysis of molecular docking results in this study included the values of Gibbs free energies of binding (ΔGbind), root-mean-square deviation (RSMD), and ligand interactions with protein residues. The ΔGbind is a thermodynamic parameter that indicates whether or not the continuation of a reaction occurs spontaneously. If the value is <0, the protein–ligand binding occurs spontaneously; if it is >0, the reaction is not spontaneous [75]. If the ΔGbind value of the tested ligand is less than that of the native ligand, it can compete with the native ligand for binding to the target receptor. On the other hand, a larger ΔGbind value indicates a less stable complex formed. RMSD indicates the average distance between the atoms (often the backbone atoms) of overlaid proteins. The smaller the RMSD, the better the model compares to the target structure. The value of each docking result was obtained from the smallest RMSD value.

The low binding free energy (BFE) value indicates that the ligand can compete for binding to the target receptor and that the resulting complex is stable. The higher a ligand’s affinity for its target protein, the more effective its activity at the cellular or organismal level. Thus, this finding indicates that iturin A has a promising antibacterial potential against *N. gonorrhoeae*. However, iturin has not been discovered in *B. cereus* to date. According to Cob-Calan et al. [76], fengycin and iturin A have a binding energy of −7 kcal/mol to β-tubulin, indicating that they have antifungal potential. Sur et al. [77] discovered that fengycin is more likely to form stable oligomers in fungal membranes than in bacterial membranes.

Non-bonded interactions (e.g., van der Waal interactions) generally contribute to a more stable protein–ligand complex and thus greater antimicrobial activity [78]. Furthermore, hydrogen bonding and hydrophobic interactions played an important role in the ligands’ binding to the receptors [79]. In the present study, the interactions that occur between the ligands and the amino acid residues of the receptors are formed as hydrogen bonds, hydrophobic interactions, and electrostatic interactions. Hydrophobic interactions occur via alkyl/pi-alkyl bonds, whereas electrostatic interactions occur via van der Waals bonds. Electrostatic interactions are salt bridges, i.e., salt bonds between oppositely charged groups in the amino acid side chain and ligand groups. A van der Waals interaction is a relatively weak electric attraction caused by molecular polarity that is either permanent or induced [80].

It is noteworthy that iturin A forms more hydrogen bonds with both PBP1 and PBP2 than any of the other compounds investigated in this present study. As more hydrogen bonds are formed with amino acid residues, it appears that the BFE value has been reduced accordingly. As a result, stronger bonds and more stable interactions were formed. Hydrophobic interactions also play a role in determining the stability of the ligand to the receptor [81]. Hydrophobic interactions are those that occur outside of a liquid environment and tend to cluster together in the globular structure of proteins [82]. The residues involved in hydrophobic interactions are nonpolar amino acid residues. Nonpolar (hydrophobic) amino acid residues tend to form clusters in the interior of the protein [83].

Molecular docking is advantageous as a first step in the development and design of new drugs because it predicts the ligand’s binding to the target protein, allowing for the determination of the receptor complex’s affinity for the ligand. The current in silico study demonstrated that fengycin and surfactin have the greatest potential as lead compounds against *N. gonorrhoeae*. MD simulations were used to get a better understanding of the interaction between proteins and ligands, to establish the spatial orientation of the receptor active site, to determine the dynamics of amino acid residues in the active site, and to evaluate the receptor’s conformational dynamics. Molecular dynamics simulation enables a more precise estimation of the thermodynamics and kinetics of recognition and binding of ligands to receptors. Additionally, these simulations accurately reproduce the behavior of the receptors at the atomic level and with extremely high temporal resolution [84].

To validate and control the created molecular dynamics system, ligand-free PBP2 was simulated under the same conditions as protein–ligand complexes. Thus, possible changes caused by fengycin and surfactin with PBP2 were analyzed. The stability of PBP2-surfactin and PBP2–fengycin complexes was demonstrated by RMSD, Rg, and RMSF trajectory analysis. The RMSD value provides information on the stability of the protein, the Rg value on its compactness, and the RMSF on its fluctuations. The complex structure will be more stable if the RMSD and Rg values are smaller and remain consistent over time. The BFE calculations based on the MM-PBSA method have been widely utilized to simulate molecular recognition because they are not only efficient but also provide insight into the interactions between ligands and receptors [85,86].

The design of drug molecules aims to find ligands that can interact effectively with target receptors [87]. This does not mean that the compound will be immediately active when administered orally. There are pharmacokinetic processes that a drug molecule must undergo in order to reach its target. These processes include absorption, distribution, metabolism, and excretion (ADME) [88]. Membrane permeability will decrease for drugs with a molecular weight greater than 1000 g/mol [89]. This should be considered when developing lipopeptides as oral medications, as iturin A, surfactin, and fengycin all have a molecular weight greater than 1000 g/mol.

The chemical ADME, including toxicity, is an important factor in the discovery and development of new drugs. The evaluation of the pharmacokinetic and toxicological properties of the evaluated compounds was therefore carried out in order to provide assurance regarding the proficiency and safety of these compounds. It appears that even the antibiotics that have been recommended to treat gonorrhea infections are not fully Ro5 compliant. However, strict adherence to the Ro5 may limit the development of natural products as drug candidates, whereas there are opportunities for developing new drugs beyond the Ro5 [90]. Many strategies to reduce the toxicity and metabolism of potential drug candidates can, on the other hand, be implemented through the decision-making process.

## 4. Materials and Methods

### 4.1. Isolation and Purification of Bacteria from Honeybee Gut

The following procedure was based on Lombogia et al. [51]. The gut of *A. dorsata* was aseptically removed and then placed in Eppendorf tubes containing sterile physiological solution (NaCl 0.95%) and homogenized using a sterile micropestle. The tube was centrifuged at 6000 rpm for 5 minutes to precipitate intestinal debris. A total of 100 μL of supernatant was taken and poured onto MRS (deMann Rogosa Sharpe) agar supplemented with 1% CaCO_3_, then incubated for 2 × 24 h at 370 °C. Colonies that grew and developed a halo zone around them reached a certain size and appeared to be distinct were then separated, and purified using a streak method. To facilitate subsequent testing, pure bacterial isolates were stored in nutrient agar (NA) slants.

### 4.2. Preparation of Indicator Bacterium

The indicator bacterium, *N. gonorrhoeae* (Zopf) Trevisan 49926™ (strain 76.061782), was purchased from a local authorized laboratory provider in lyophilized form. The following procedure was a modification of Sanders’ [91]. The bacterium was inoculated in nutrient broth (NB), then incubated at 37 °C for 1 × 24 h to revive the bacterium. Following visible growth, 0.1 mL of the culture was inoculated into nutrient agar (NA), which was then evenly spread with L-glass and incubated for 1 × 24 h at 37 °C. Following that, the indicator bacterium was reinoculated into the NA slant. It was then incubated at 37 °C for 24 h and was ready for further testing.

### 4.3. Antibacterial Test of Gut-Associated Bacteria

Pure gut-associated bacterial isolates recovered from *A. dorsata* were then tested for their antibacterial activity against bacterial indicator *N. gonorrhoeae* using the agar well diffusion method, following the previous method by Lombogia et al. [51]. Prior to testing, the indicator bacterium was measured for turbidity following the McFarland turbidity standard [92]. The indicator bacterium was then pipetted up to 500 μL into an Erlenmeyer flask containing 50 mL of nutrient agar.

A total of 10 mL of NA was poured into a Petri dish containing four stainless-steel cylinders to form wells [93]. Following the hardening of the media, another 10 mL of NA was added, which had been mixed with the indicator bacteria *N. gonorrhoeae*. After allowing the media to harden, the stainless-steel cylinders were removed to create wells.

The subsequent procedure was similar to that described by Yelnetty et al. [53]. Each gut-associated bacterial isolate was first grown for 24 h at 37 °C in an Eppendorf tube containing NB. After incubation, the bacteria were heat-killed in a thermo-block at 80 °C for 1 h (treatment 1). Two Eppendorf tubes with killed bacteria were set aside for vortexing, whereas the other two tubes were not. Additionally, the four tubes were centrifuged for 1 min at 6000 rpm to obtain cell-free culture supernatants (CFCS). Each well received 100 μL of each CFCS. An amount of 30 μg/mL doxycycline was used as a positive control and sterile ddH_2_O as a negative control. The diameter of the inhibition zone produced by gut-associated bacterial isolates was measured in Petri dishes over a three-day period at 37 °C. The diameter of the inhibition zone was measured using a ruler on a daily basis. The presence of a clear zone around the well characterizes this inhibition zone. In addition, non-heated supernatants were neutralized with NaOH to achieve a pH of 6.0 (treatment 2). This was intended to neutralize organic acids and to predict the antimicrobial peptides that were likely produced by isolates [93]. Evaluation of test results was based on classification of inhibition by Zare Mirzaei et al. [94] as follows: <11 mm (negative), 11–16 mm (+ weak), 17–22 mm (++ strong), dan > 23 mm (+++ very strong).

### 4.4. Molecular Identification of Bacterial Isolates

Purified bacterial isolates with antibacterial activity were identified molecularly using the 16S rRNA marker gene, as described by Fatimawali et al. [95].

### 4.5. In Silico Analysis of Antibacterial Potential by Molecular Docking Method

The antibacterial compounds (surfactin, fengycin, and iturin A) examined in this study were identified through a review of several articles on *B. cereus* antimicrobial lipopeptides. According to the literature, these lipopeptides were found in *Bacillus* [96,97]. Surfactin and fengycin were discovered in the acidified cell-free culture filtrate (CFCF) of *B. cereus* TSH77 [37]. *B. cereus* was chosen based on the findings of this study, as described in the results and discussion. Molecular docking studies were performed using the iGEMDOCK version 2.1 software [98]. The crystal structures of penicillin-binding protein (PBP) 1 and 2 were downloaded from the RSCB protein data bank website under the PDB IDs 5TRO (resolution: 1.80 Å) and 6VBC (resolution: 1.55 Å), respectively, and saved for subsequently uploaded to the iGEMDOCK. The 5TRO is a dimerization and transpeptidase domain (residues 39–608) of Penicillin-binding Protein 1 from *Staphylococcus aureus*, while the 6VBC is a transpeptidase domain of PBP2 from *N. gonorrhoeae* cephalosporin-resistant strain H041. The missing residues of 5TRO were completed with SWISS-MODEL [99]. The structures of surfactin (CID 65307), fengycin (CID 62705048), and iturin A (CID 102287549) were obtained from the PubChem website (http://pubchem.ncbi.nlm.nih.gov, accessed on 12 November 2021). The ligands’ minimized 3D structures were prepared in ChemDraw3D v.19.0 and saved as mol2 files. After loading prepared ligands and binding site, the docking was initiated in Standard Docking accuracy settings. The results were recorded and analyzed. Ligands’ binding poses and protein-ligand interactions were demonstrated with Chimera v.1.15 [100] and Discovery Studio Visualizer v2021.

### 4.6. Molecular Dynamics Simulation

MD simulations were conducted using the Gromacs v. 2019.4 [101,102,103] after attaining the required conformation via docking. Using the gromos54a7 [104,105] force field, the protein PBP2 topologies were constructed in Gromacs using the pdb2gmx module. The water molecules were modeled with SCP [106,107], and then ions were added. The GlycoBioChem PRODRG2 server was used to construct the ligand topologies [108]. The protein complexes were placed at least 1.0 nm from the box edge in a dodecahedron box. To neutralize the charge systems, sodium ions were introduced. The energy of the simulation system was minimized by performing 50,000 steps of the steepest descent minimization algorithm. Two constrained phases were used to equilibrate the solvent and ion systems. The canonical ensemble of NVT (mol (N), volume (V), and phase equilibrium temperature (T) of the system was performed at 300 K by using the V-rescale method [109] with a duration of 0.3 ns. The Parrinello–Rahman method [110] was used to perform the isothermal-isobaric ensemble NPT (moles (N), pressure (P), and temperature (T) equilibrium phase at 0.3 ns under 1 atm of pressure. A leapfrog MD integrator was used to create 1000 frames with a length of 100 ns. Finally, the root-mean-square deviation (RMSD), root-mean-square fluctuation (RMSF), and radius of gyration (Rg) trajectory analyses were conducted.

### 4.7. MM-PBSA Binding Free Energy Calculation

The BFE calculation based on molecular mechanics and Poisson–Boltzmann surface area (MM-PBSA) is frequently used to determine the stability and bonding strength of protein–ligand, protein–peptide, and protein–protein complexes [111]. The calculation of BFE for the ligand-receptor complexes was performed in this study by utilizing the MM-PBSA method using 50 frames spanning 80–100 ns from the MD trajectory. The average BFE calculations were performed using the ‘MmPbSaStat python’ script integrated in g_mmpbsa [111,112].

### 4.8. Lipinski’s Rule of Five

The drugability of surfactin, fengycin, and Iturin A was analyzed based on the criteria determined by Lipinski’s rule of five (Ro5) [113]. Information on this was obtained from the supercomputing facility for Bioinformatics and Computational Biology, IIT Delhi (http://www.scfbio-iitd.res.in/software/drugdesign/lipinski.jsp, accessed on 12 November 2021).

### 4.9. ADMET Analysis

The pharmacokinetic properties and druglike nature of the compounds were evaluated by predicting ADME parameters using SwissADME (http://www.swissadme.ch/, accessed on 12 November 2021) [114] and pkCSM (http://biosig.unimelb.edu.au/pkcsm/, accessed on 12 November 2021) (Pires et al., 2015). Toxicology predictions were made using ProTox-II (https://tox-new.charite.de/protox_II/, accessed on 12 November 2021) [115]. The canonical SMILES of the compounds used as input were obtained from the PubChem database.

## 5. Conclusions

The current study examines the ability of components produced by *B. cereus* for the control of gonococcal disease. Our in vitro investigation revealed that the cell-free supernatant of *B. cereus* isolated from the gut of *A. dorsata* has antibacterial activity, inhibiting the growth of *N. gonorrhoeae*. As determined by a literature study, *Bacillus* sp. produces the lipopeptides surfactin, fengycin, dan iturin A. According to our results of in silico research utilizing a molecular docking method, these three lipopeptides exhibited binding free energy values comparable to those of the antibiotics ceftriaxone, cefixime, and doxycycline against the target protein receptors of *N. gonorrhoeae*, PBP 1 and PBP2. Surfactin displays high stability when interacting with PBP2 of *N. gonorrhoeae*, despite alterations in hydrogen bonding and hydrophobic interactions, according to our molecular dynamic modeling studies. As a result, surfactin has a promising future as an anti-gonorrhea agent. The study’s limitation is that it cannot be conclusively established that surfactin, fengycin, and iturin A all play a role in inhibiting the growth of *N. gonorrhoeae*, as their presence is based on assumptions. It is, therefore, strongly recommended that these findings be validated by isolating lipopeptides from *Bacillus* sp., particularly surfactin, and testing their ability to inhibit the growth of *N. gonorrhoeae* in vitro.

## Figures and Tables

**Table 1:** Results of antibacterial activity analysis of *A. dorsata* gut-associated bacterial isolates against *N. gonorrhoeae*.

Isolate Code	Average Diameter of Inhibition Zone (mm ± S.D.)	
	Treatment 1	Treatment 2
LJ1	9.50 ± 0.50	11.33 ± 0.29
LJ2	17.18 ± 0.29	22.33 ± 0.29
LJ3	14 ± 0.50	16.33 ± 0.29
LJ4	18.33 ± 0.29	24.17 ± 0.29
LJ5	21.83 ± 0.29	21.83 ± 0.29
LJ6	23.33 ± 0.29	23.83 ± 0.29
LJ7	18.33 ± 0.29	21.17 ± 0.29
Positive control	26.33 ± 0.29	26.50 ± 0.29
Negative control	0	0

**Table 2:** Molecular identification of *A. dorsata* gut-associated bacterial isolates using 16S rRNA gene markers.

Isolate Code	Species	% Identity
LJ1	*Bacillus anthracis*	99.92
	*B. thuringiensis*	99.92
	*B. cereus*	99.92
	*B. tropicus*	99.92
	*B. paramycoides*	99.92
LJ2	*Acinetobacter indicus*	100
LJ3	*B. cereus*	100
LJ4	Noise sequence result	-
LJ5	Noise sequence result	-
LJ6	*B. cereus*	100
LJ7	*B. cereus*	100

**Table 3:** Molecular docking analysis between surfactin, fengycin, and iturin A with receptors PBP 1 and 2.

Ligands	PubChem CID	Binding Free Energy (kcal/mol)	
		PBP1 (PDB ID: 5TRO)	PBP2 (PDB ID: 6VBC)
Ceftriaxone	5479530	−117.49	−137.87
Cefixime	5362065	−105.15	−113.39
Doxycycline	54671203	−104.23	−113.13
Fengycin	62705048	−103.21	−114.55
Surfactin	65307	−118.37	−103.74
Iturin A	102287549	−124.42	−127.67

**Table 4:** Analysis of the compounds’ interactions with receptor PBP 1.

Compounds	Number of H-Bonds	Interacting Residues with Hydrogen Bonds
Ceftriaxone	7	Conventional H-bond: Asn:A118, Asn:A144, Ile:A117, Ser:A114; Carbon H-bond: Asp:A149 (2), Asn:A144; Alkyl/Pi-Alkyl: Leu:A145, Arg:A140.
Cefixime	4	Conventional H-bond: Arg:A504, Ser:A590; Carbon H-bond: Asp:A506, Asn:A494; Alkyl: Ala:A501, Arg:A504; Sulfur-X: Arg:A504
Doxycycline	4	Conventional H-bond: Lys:A545. Glu:A486, Glu:A483, Asp:A480; Carbon H-bond: Glu:A483; Alkyl/Pi-Alkyl: Lys:A545; Unfavorable Acceptor-Acceptor: Glu:A486, Glu:A483
Fengycin	4	Conventional H-bond: Gln:A518, Glu:A311, Ser:A426; Carbon H-bond: Tyr:A527; Alkyl: Ala:A520,Ala:A406
Surfactin	4	Conventional H-bond: Ser:A349, Thr:A516,Ser:A314; Carbon H-bond: Trp:A351; Alkyl: Ile:A348
Iturin A	9	Conventional H-bond: Thr:A309 (2), Asn:A308 (2),Trp:A301,Asp:A267, Lys:A300; Carbon H-bond: Asn:A308, Lys:A266; Alkyl/Pi-Alkyl: Ala:A521 (2), Lys:A266, Val:A528, Pro:A522 (3), Trp:A301, Ala:A302

**Table 5:** Analysis of the compounds’ interactions with receptor PBP 2.

Compounds	Number of H-Bonds	Interacting Residues with Hydrogen Bonds
Ceftriaxone	9	Conventional H-bond: Ser:A545, Thr:A500 (2), Ser:A310, Asn:A364 (3), Thr:A347 (2); Carbon H-bond: Ser:A310; Pi-Cation: Lys:A313
Cefixime	6	Conventional H-bond: Tyr:A544 (2), Ser:A362; Carbon/Pi-Donor H-bond: Ser:A483, His:A348; Pi-Lone Pair: Lys:A361; Pi-Sulfur: His:A348; unfavorable bump: His:A348
Doxycycline	8	Conventional H-bond: Phe:A492, Val:A489 (2), Asp:A490, Thr:A573, Gly:A491 (2), Pro:A571; Pi–Alkyl: Lys:A570; unfavorable Donor-Donor: Pro:A571, Lys:A570
Fengycin	4	Conventional H-bond: Arg:A528, Pro:A522; Carbon H-bond: Arg:A528, Pro:A456; Alkyl/Pi-Alkyl: Arg:A271, Arg:A528, Leu:A564; unfavorable bump: Thr:A272
Surfactin	4	Conventional H-bond: Thr:A343 (2), Gln:A345; Carbon H-bond: Thr:A343
Iturin A	11	Conventional H-bond: Asn:A364 (2), Phe:A420, LysA313 (2), Ser:A310 (2), Ser:A362, Thr:A347, Tyr:AA422; Carbon H-bond: Tyr:422; Pi-Alkyl:Tyr:A543, Tyr:A544

**Table 6:** MM-PBSA binding free energies of PBP2 with compounds surfactin and fengycin between 80 ns and 100 ns.

Parameters (Energy)	Protein–Ligand Complexes	
	PBP2–Surfactin (kJ/mol)	PBP2–Fengycin (kJ/mol)
Van der Waals	169.951 ± 15.249	−177.548 ± 16.375
Electrostatic	−20.419 ± 12.130	−41.944 ± 19.656
Polar solvation	82.717 ± 19.749	121.842 ± 55.225
SASA	−16.912 ± 1.643	−17.907 ± 3.294
Binding free	124.564 ± 13.713	−115.557 ± 44.567

**Table 7:** Lipinski’s Ro5 analysis of fengycin, surfactin, and iturin A.

Compounds	Molecular Formula	Lipinski’s Parameters				
		Molecular Weight (<500 Da)	LogP (<5)	H-Bond Donor (<5)	H-Bond Acceptor (<10)	Violations
Fengycin	$C_{72}H_{110}N_{12}O_{20}$	1463.71	1.36	16	21	3
Surfactin	$C_{53}H_{93}N_{7}O_{13}$	1036.34	4.00	9	13	3
Iturin A	C_48_H_74_N_12_O_14_	1043.2	−1.8	13	14	3

**Table 8:** ADMET analysis of each compound.

Parameters	Ceftriaxone	Cefixime	Doxycycline	Fengycin	Surfactin	Iturin A
Molecular weight (g/mol)	554.6	453.5	444.4	1463.7	1036.3	1043.2
H-bond acceptor	13	12	9	21	13	14
H-bond donor	4	4	6	16	9	13
CNS	−4.149	−4.079	−3.958	−5.703	−2.326	−5.459
CYP2D6 substrate	No	No	No	No	No	No
CYP3A4 substrate	No	No	No	Yes	Yes	No
CYP1A2 inhibitor	No	No	No	No	No	No
CYP2C19 inhibitor	No	No	No	No	No	No
CYP2C9 inhibitor	No	No	No	No	No	No
CYP2D6 inhibitor	No	No	No	No	No	No
CYP3A4 inhibitor	No	No	No	No	No	No
Carcinogenicity	No	No	No	No	No	No
Hepatotoxicity	Yes	Yes	Yes	No	Yes	No
P-glycoprotein substrate	No	No	Yes	Yes	Yes	Yes
Acute oral toxicity	Class VI	Class VI	Class IV	Class V	Class IV	Class IV

## Data Availability

Data is included in this article. Additional data regarding this article will be provided upon request.
